# Teaching medical students how to interact with the pharmaceutical industry: A scoping review

**DOI:** 10.3205/zma001578

**Published:** 2022-11-15

**Authors:** Samiyah Farah, Justin L. Bilszta

**Affiliations:** 1 University of Melbourne, Melbourne Medical School, Department of Medical Education, Melbourne, Australia

**Keywords:** education, medical, undergraduate, curriculum, medical students, drug industry

## Abstract

**Objectives: **The influence of the pharmaceutical industry is of significant concern in physician prescribing decisions; medical students may not be fully equipped with the knowledge or skills to manage interactions with industry prior to graduation. The aim of this study was to evaluate the characteristics of educational interventions undertaken to improve students’ knowledge, attitudes, and skills in managing interactions with the pharmaceutical industry.

**Methods: **A systematic search of Ovid Medline, EMBASE, CINAHL and ERIC databases identified 3210 primary studies with keywords related to “pharmaceutical industry” and “undergraduate medical education”. Eleven articles were included for review.

**Results: **Disparate methods of teaching medical students how to interact with the pharmaceutical industry were identified, making it difficult to compare the effectiveness of different educational interventions. All the included studies achieved the aims of the described intervention, at least in the short term, suggesting perhaps any education related to interactions with the pharmaceutical industry can aid students in managing these situations.

**Conclusions:** The lack of an evidence-base means more research into the identification of educational interventions which engender durable changes in students' knowledge, attitudes, and skills to manage interactions with the pharmaceutical industry are required. Any intervention will likely be context-dependent, as a universal approach is hindered by the fact different countries have different laws governing pharmaceutical industry-physician interaction.

## Zusammenfassung

**Ziele: **Der Einfluss der pharmazeutischen Industrie ist von erheblichem Belang für die Verschreibungsentscheidungen von Ärzt*innen. Medizinstudierende werden vor ihrem Abschluss möglicherweise nicht vollumfänglich mit den nötigen Kenntnissen oder Kompetenzen für die Interaktion mit der Industrie ausgestattet. Ziel dieser Studie war die Evaluation der Charakteristika von Lehrinterventionen zur Verbesserung der Kenntnisse, Verhaltensweisen und Kompetenzen der Studierenden im Umgang mit der pharmazeutischen Industrie.

**Methoden: **Bei der systematischen Durchsuchung der Datenbanken Ovid Medline, EMBASE, CINAHL und ERIC wurden 3210 Primärstudien mit den Suchwörtern „pharmaceutical industry“ (pharmazeutische Industrie) und „undergraduate medical education“ (Medizinstudium) identifiziert. Elf Artikel wurden in den Review eingeschlossen.

**Ergebnis: **Die Recherche ergab, dass die beschriebenen Methoden der Ausbildung von Medizinstudierenden in der Interaktion mit der pharmazeutischen Industrie sehr unterschiedlich waren, weshalb es schwierig ist, die Wirksamkeit der verschiedenen Lehrinterventionen zu vergleichen. Alle eingeschlossenen Studien erreichten die Ziele der beschriebenen Intervention zumindest kurzfristig, was nahelegt, dass möglicherweise jede Bildungsmaßnahme zur Interaktion mit der pharmazeutischen Industrie für Studierende hilfreich sein kann im Umgang mit diesen Situationen.

**Fazit: **Der Mangel an Evidenzgrundlagen bedeutet, dass weitere Forschung nötig ist, um Lehrinterventionen zu identifizieren, die nachhaltige Veränderungen der Kenntnisse, Verhaltensweisen und Kompetenzen der Studierenden in der Interaktion mit der pharmazeutischen Industrie bewirken. Die Intervention wird wohl von den jeweiligen Bedingungen abhängig sein, da einem universellen Ansatz die Tatsache entgegensteht, dass für Interaktionen zwischen Pharmaindustrie und Ärzt*innen in verschiedenen Ländern verschiedene Gesetze gelten.

## Introduction

Few topics in the world of medicine are as fervently debated as the interactions between the pharmaceutical industry and physicians [1], [2]. A major concern is how pharmaceutical industry-physician relationships influence patient care, and whether considerations of cost and medication efficacy are outshined by effective marketing techniques [3], [4], [5], [6]. Available evidence suggests policy and educational interventions can be effective in modifying attitudes towards pharmaceutical industry interactions, as well physicians' prescribing behaviour [7] However, data on the long-term effects of these interventions are limited [8], [9], [10].

Given this degree of concern in the physician space, it is important to consider what medical schools are doing to ensure adequate teaching on how interactions with the pharmaceutical industry may impact their graduates, as patterns learned in medical school may well influence future behaviour [11]. *The Parliamentary Assembly, Council of Europe (PACE) has called for member states to “…incorporate, into the curriculum for health care professionals specific, mandatory training to foster awareness of the influence of pharmaceutical promotion and how to respond…”* [12]. The World Health Organization’s Ethical Criteria for Medicinal Drug Promotion [13] outlines steps to support and encourage the improvement of health care through the rational use of medicinal drugs. It addresses a range of drug promotion activities including advertisements; [the role of] medical representatives; free samples of prescription and non-prescription drugs for promotional purposes; symposia and other related scientific meetings; packaging and labelling information for patients. 

Despite widespread promotion of the PACE recommendation, and acceptance of the WHO Criteria, many medical students report feeling ill-prepared to manage inevitable interactions with industry once they graduate as doctors [11]. *The reasons for this are unclear; it may be because the PACE and WHO directives don’t specifically provide a clear explanation of how they apply to the medical student context only referring to their use in “…curriculum for health care professionals…”, “…universities and other teaching institutions…” or those “…[health personnel] involved in the prescription, dispensing, supply and distribution of drugs…”* [12], [13]. *In both instances, there is no guidance on how undergraduate medical education providers might be able to incorporate these requirements into their curricula***.** It is clear without satisfactory education, students may accept pharmaceutical promotional materials at face value, without sufficient awareness of potential biases interaction with the industry can engender [14].

In the medical literature, significant attention has been given to evaluating medical student attitudes towards, and the evaluation of the level of interaction students have with, the pharmaceutical industry. Austad et al., in their systematic review of studies evaluating the frequency and nature of medical student’s exposure to the drug industry, concluded 70-100% of clinical year medical students around the world have had some form of interaction with the pharmaceutical industry, with the highest levels of exposure occurring in the USA [15]. Most students purported the acceptance of gifts as ethically permissible, stating reasons such as financial difficulty and societal norms in that the majority of other students also accept gifts, making it acceptable on an individual level [15]. Alarmingly, Austad et al. found almost 2/3rds of students consider themselves immune to bias in the acceptance of gifts and interactions with pharmaceutical representatives [15]. These findings raise the question: how should medical students be taught to navigate interactions with the pharmaceutical industry? A review of educational interventions in medical schools regarding how to critically engage with the industry and how these interventions impact student behaviour, if at all, is lacking in the literature.

We, therefore, conducted this scoping review of primary studies to identify the characteristics of educational interventions that aim to improve medical students’ attitudes, knowledge, and skills when interacting with the pharmaceutical industry. The specific research questions we sought to answer were:

1. how do medical schools teach students about how to interact with the pharmaceutical industry? and;
2. to what extent do such educational interventions impact medical student attitudes, knowledge, and skills regarding industry?

In addition to these questions, we evaluated the effectiveness of interventions described in the included studies using the Kirkpatrick Hierarchy for Assessing Educational Outcomes [16], [17].

## Methods

This study implemented the “Preferred Reporting Items for Systematic reviews and Meta-Analysis Extension for Scoping Reviews (PRISMA-ScR)” reporting protocol [18].

### Search strategy

Online biomedical, pharmacological and education databases EMBASE, Ovid Medline, CINAHL and ERIC were searched for primary studies describing educational interventions that address medical student interactions with the pharmaceutical industry. The reference list of each included full text articles was also searched for additional studies not identified through the database search.

#### Inclusion and exclusion criteria

The search was not limited by publication date. *Only full text articles written in English were considered for inclusion.* The inclusion criteria were primary studies reporting on targeted educational interventions designed to assist medical students in managing interactions with the pharmaceutical industry. Articles which only reported on medical student attitudes towards the pharmaceutical industry and/or numerical quantification of interactions between students and pharmaceutical representatives, were excluded. Literature reviews (narrative or systematic) were excluded, as were conference abstracts. Also excluded were studies which evaluated the impact of policies limiting medical student interaction with the pharmaceutical industry if there was no associated educational intervention.

#### Key terms and boolean operators

The key search terms and boolean operators used are described in table 1 (Tab. 1).

#### Data extraction and synthesis of results

Data extraction was performed using a predetermined list and included: author and year; participant characteristics including location of study; aims of the study; description of the educational intervention; background of the instructors; duration of the intervention; methods utilised to measure the outcomes of the intervention; timing of outcome measurement; main findings, and; study limitations.

**Figure 1:**
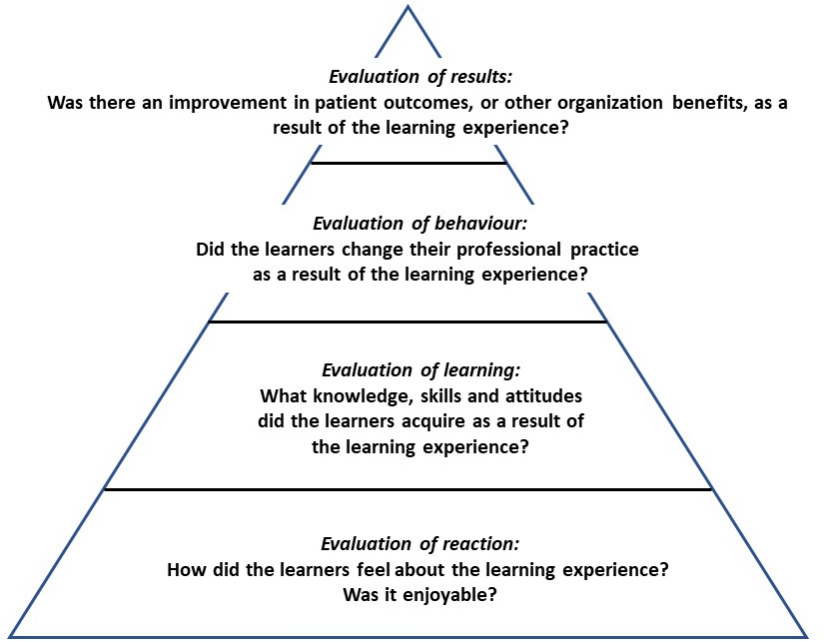
Kirkpatrick ’s Hierarchy of Educational Outcomes

The impact of the educational outcomes was assessed using Kirkpatrick's Hierarchy of Educational Outcomes (see figure 1 (Fig. 1)), a well-recognised tool for the evaluation of effectiveness of medical educational outcomes [16], [17]. The first level assesses learners’ satisfaction with, or reaction to, the intervention; the second level assesses modification of students' attitudes and perceptions and/or the knowledge and skills learned; the third level assess changes in health professionals' behaviour or an institution's practice, and; the fourth level assess changes in patient health care outcomes.

#### Synthesis of results

Included studies were described by the characteristics listed above. Thematic analysis was conducted to identify commonality between included studies. No inferences were made about teaching, learning and assessment approaches if they were not explicitly stated.

Literature searching, title and abstract screening, full text review and data extraction, and charting were undertaken by the 1^st^ author (SF). Where there was any uncertainty regarding the aforementioned, these articles were reviewed independently by the 2^nd^ author (JB) and then discussed until consensus was reached between both authors. The 2^nd^ author also independently reviewed the data extraction and charting results once this process was completed by the 1^st^ author.

## Results

**Figure 2:**
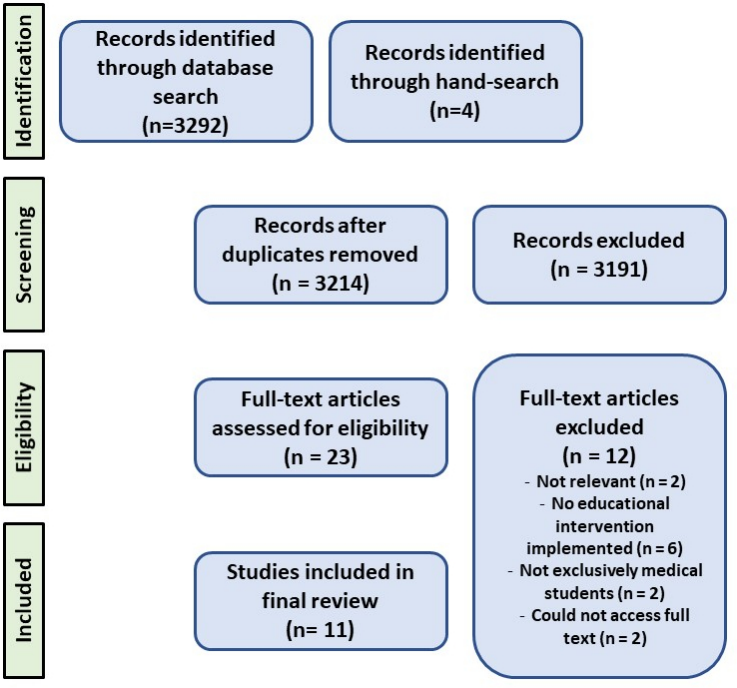
PRISMA diagram

*The database search was conducted between July-August 2021 and identified 3296 articles (see figure 2* (Fig. 2)*) – 129 from CINAHL, 44 from ERIC, 310 from Ovid Medline, 2809 from EMBASE and 4 from hand-searching of reference lists. After removal of duplicates, 3214 articles remained. *Following title and abstract screening, 23 articles remained for full text review. Full text review yielded 11 articles that met the inclusion criteria.

Of the 11 studies included, the majority were conducted in the USA [11], [14], [19], [20], [21], [22], as well as Turkey [23], Nepal [24], [25] and India [26], [27]. Details of included studies are summarised in attachment 1 .

### Types of interventions

The structure of delivery varied widely amongst the studies. Four studies [11], [19], [20], [23] used an intervention and control group with students of different year levels whilst the remaining studies [14], [21], [22], [24], [25], [26], [27] delivered the intervention to a single cohort of students. Almost all programs comprised of students in either their 2^nd^ or 3^rd^ year of study, though course duration varied. Two studies [14], [21] made the educational interventions compulsory, five non-compulsory [22], [23], [24], [25], [26], [27], and four [11], [19], [20], [26] were not stated. Kao et al [11] was the only study which delivered an intervention in multiple institutions; all other studies assessed students at a single institution.

The duration of the interventions differed across the studies; teaching time ranged from 40 minutes [20] to 14 hours [26]. Two programs adopted an extended approach where, rather than a single time delivery, teaching was delivered regularly over a period of 4 months [24], [25].

Teaching modality varied across interventions; some utilised a didactic presentation followed by an instructor led discussion [14], [19], [26], [27] whilst others [14], [23], [24], [25] used role plays to demonstrate a typical encounter with a pharmaceutical representative, in addition to lectures and/or other teaching modalities. In two studies [26], [27] students were assigned pharmaceutical promotion materials, such as articles and brochures, to critique. Four [11], [23], [24], [25] interventions incorporated numerous teaching modalities; for example, Shankar et al. [24], [25] employed instructor presentations, brainstorming sessions, group activities and role plays. Corbin et al. [22] developed a series of videos to educate students on the influence of pharmaceutical marketing techniques on evidence-based prescribing. Markham et al. [20] described an approach whereby students were assigned articles to summarise and present to other students followed by a discussion facilitated by a faculty member.

Most interventions were delivered by medical school faculty members [11], [14], [19], [20], [21], [24], [25] with the remaining utilising pharmaceutical industry representatives [11], [21] in addition to faculty. Civaner et al. [23] recruited a specialist in marketing education, as well as a pharmaceutical representative. The intervention described by Wilkes et al. [14] was delivered by pharmacists, one of whom was a former pharmaceutical representative. Three studies [22], [26], [27] did not report the background of the instructors.

#### Intervention aims and outcome measures 

Generally, the aims of each intervention fell into two broad categories: those that aimed to improve students’ ability to critically appraise promotional literature [24], [25], [26], [27] or those evaluating the impact of their intervention on students’ attitudes and knowledge on the effects of industry influence [11], [14], [20], [21], [22], [24]. Variations to this were noted. Vinson et al. [19] explored medical student attitudes, pre and post the intervention, towards the acceptance of gifts from the pharmaceutical industry. Civaner et al. [23] evaluated the durability, throughout the students’ clinical years, of an educational intervention focused on students’ attitudes regarding industry promotional strategies.

Six studies [11], [14], [19], [20], [21], [23] utilised pre and post intervention surveys to measure changes in knowledge and attitudes on the interface between the pharmaceutical industry and physicians, such as opinions about marketing tactics and the acceptance of gifts. Of note, Corbin et al. [22] administered knowledge-based tests to objectively assess students' knowledge and skills of pharmaceutical marketing techniques, while Nayak et al. [27] and Sayyad et al. [26] used a similar approach to assess student’s skills in identifying violations of WHO criteria in drug promotional materials. The remaining two studies [24], [25] administered post-intervention surveys only.

Four [11], [19], [20], [23] studies compared the results of the post-intervention survey assessing the impacts of their respective interventions to a cohort of students not exposed to the intervention. In each of these studies, the intervention groups were more likely to change their attitudes towards pharmaceutical marketing practices and/or gain knowledge regarding the influence these companies can have on prescribing decisions, compared to the unexposed group. The remaining studies [14], [21], [22], [24], [25], [26], [27] evaluated each student’s survey responses pre and post intervention. Overall, all included studies demonstrated their interventions were effective in improving students’ knowledge and skills, and/or changed their attitudes regarding the pharmaceutical industry, either in comparison to the group not exposed to the educational intervention or compared to the individual students’ pre-intervention surveys.

The studies varied in the timing of the post intervention outcome measure. Three studies administered the post intervention survey between 1 [11], [26] and 4 years [23] following the intervention. Three studies [14], [19], [20] administered the post-intervention survey 6-12 weeks following the intervention, two studies [21], [27] immediately after, and three [22], [24], [25] did not specify the timing of the post-intervention measure. Of the three studies which assessed students at least 1-year post-intervention, two [11], [26] noted the intervention had a sustained effect on the students’ knowledge or attitudes towards the industry. Kao et al. [11] reported students were more likely to hold the view pharmaceutical marketing practices exert moderate-to-strong influences on prescribing decisions. Sayyad et al. [26] demonstrated significant improvement in students’ ability to recognise violations of WHO guidelines on drug advertisements a year after the educational program. The last of these studies [23] reported students were less likely to hold the opinion *“…it is important to avoid financial incentives from pharmaceutical companies given the influence this can have on prescribing habits…”* four years after the intervention, in comparison to immediately after the intervention.

#### Assessment of learning outcomes

Kirkpatrick’s hierarchy of educational outcomes was used to assess the learning outcomes of each intervention and this is presented in attachment 1 . Of note, only one intervention [11] achieved level 3 criteria, wherein students applied or planned to apply the knowledge gained from the intervention. No intervention achieved level 4 criteria, whereby improvements in patient care could be correlated with the educational intervention. All other interventions achieved either level 1 or level 2 criteria.

## Discussion

It is well established effective marketing by pharmaceutical industry can influence physician prescribing habits, which may not be in the best interests of patients [3], [4], [28], [29]. Students may be ill-prepared to recognise the power of advertising and promotional strategies on their future prescribing behaviours [20]. It is imperative, therefore, medical schools intervene to ensure their graduates enter their professional careers equipped with the knowledge and skills to interact appropriately with the pharmaceutical industry. The following discussion addresses the key findings and implications of the current study.

The literature in this space proved to be sparse with only 11 studies identified for inclusion. Each of the included studies described interventions with different aims, pedagogical designs, student cohorts, duration of teaching and instructor backgrounds, thereby making it difficult to compare interventions and make recommendations as to which educational approaches are the most efficacious.

Nonetheless, it is noteworthy each of the included studies achieved the aims of the intervention – that is, they reported a change in attitudes towards interactions with the pharmaceutical industry and/or gained knowledge and skill in interacting with the industry – whether as a single lecture-discussion [19] or a structured module delivered over an extended period [24]. *Whilst there is not yet enough evidence to demonstrate a causal relationship between an education intervention and changes in students’ attitudes, knowledge and skills, the results presented suggest* that even minimal incorporation of education focussed on pharmaceutical industry-physician interactions may *assist medical students manage these relationships*.

Of the 11 studies reviewed, only three [11], [23], [26] focused on the durability of behaviour and/or attitude change. Kao et al. [11], the study with the largest sample size and the only to assess students at multiple institutions, reported students exposed to the intervention were more likely to believe physicians are influenced by pharmaceutical marketing, compared to the control group, at least 1-year post intervention. Sayyad et al. [26] demonstrated students were able to identify violations of pharmaceutical companies in drug promotional materials even a year following the educational intervention. Lastly, Civaner et al. [23] found although students were more sceptical about pharmaceutical representatives, and became more wary about their influence on physicians post-intervention, this was subject to erosion once students were socialised into the clinical environment and observed real-world interactions between the pharmaceutical industry and more senior colleagues. This is important as students report they base their prescribing decisions on examples provided by role models [30], and has been observed in other attitudinal changes during medical school [31], [32] [33]. 

The above results highlight several important considerations: curriculum designers need to ensure any educational effects last beyond the period immediately post-intervention and withstand the influence of the clinical environment; the importance of role modelling behaviour of senior colleagues and; a structured and objective approach to help students increase their ability to critically appraise pharmaceutical company literature in a way that is sustained over time.

As aforementioned, only one study [25] achieved Kirkpatrick level 3, wherein students applied or planned to apply the knowledge gained from the intervention. None of the included studies assessed achieved Kirkpatrick level 4 meaning it was not possible to draw conclusions as to which interventions resulted in a measurable effect on patient outcomes in the long term.

An important limitation of this review is the studies were completed in a variety of different countries – Turkey [23], Nepal [24], [25], India [26], [27] and the USA [11], [14], [19], [20], [21], [22]. Although* no studies from western or central Europe were included in the analysis, this is not a result of methodological flaws. The search strategy was systematic using a comprehensive range of key words and search terms. Both authors regularly discussed and critiqued the search results, and reviewed how the inclusion and exclusion criteria were applied. The results presented accurately represent the current available evidence-base regarding educational interventions specifically focused on helping undergraduate medical students manage their interactions with the pharmaceutical industry. The studies included* are from countries vastly different government policies and organizational frameworks governing pharmaceutical industry-physician interactions. This makes generalisations related to a best-practice approach difficult, if not impossible, and suggests curricula in this area will need to be highly context-dependent. That is, in countries where there are minimal legislated limitations on industry-physician interaction, there will need to be an entirely different educational approach compared to countries where there are significant legislative limitations designed to minimise industry influence.

Several other limitations with this study are noted. Relevant articles may have been omitted due to the nature of the search strategy, including only articles written in English were assessed for inclusion. Sample sizes were small, and only one study [11] had a sizeable number of participants and assessed students at multiple institutions. *A critical evaluation of the included literature was not performed and no determination as to the quality of the evidence/outcomes reported in each included study has been made*. Numerous disparities in the nature and design of the studies creates difficulties in comparing findings. Year of publication ranged from 1993 [19] to 2020 [23]. *Studies reported findings on preclinical* [19]**, **[22]**, **[23]**, **[24]**, **[25]**, **[26]**, **[27] *and clinical* [11]**, **[14]**, **[20]**, **[21] *students with differing levels of exposure to pharmaceutical representatives*. Only two studies [14], [21] mandated completion of the educational intervention, whereas the rest [22], [24], [25], [27] were delivered on a voluntary basis, adding to the disparity in approaches. Aside from three studies [22], [26], [27] which utilized objective, exam-style questions to assess students' knowledge post-intervention, the remaining studies used qualitative surveys to evaluate changes in students’ knowledge and attitudes. Finally, several studies [22], [26], [27] did not report all the extracted data, namely instructor background.

*Pharmaceutical industry interaction with physicians occurs in a variety of different contexts including one-on-one meetings with industry representatives, industry-sponsored continuing medical education events, promotional material and industry-sponsored conferences/lectures/symposia* [4], [5]. Whilst medical students are in the formative years of training, it is essential to equip them with the knowledge and skills required to appropriately manage these interactions. This review establishes a measurable change in students’ attitudes and knowledge regarding the effect of industry influence can be elicited even after an intervention delivered at a single time point; however, further research is needed to create interventions that are durable beyond the period immediately post-intervention and withstand the influence of the clinical environment. This review also highlights that generalisations related to a best-practice approach to equipping students to navigate the complexity of pharmaceutical industry-physician relationships are difficult given the highly context-dependent policies governing such relationships. But, this provides curriculum developers the opportunity to document a diversity of teaching, assessment and evaluation approaches. This will ultimately add to our understanding of how to best empower the next generation of doctors in their dealings with the pharmaceutical industry. 

## Competing interests

The authors declare that they have no competing interests. 

## Supplementary Material

Data extraction

## Figures and Tables

**Table 1:**
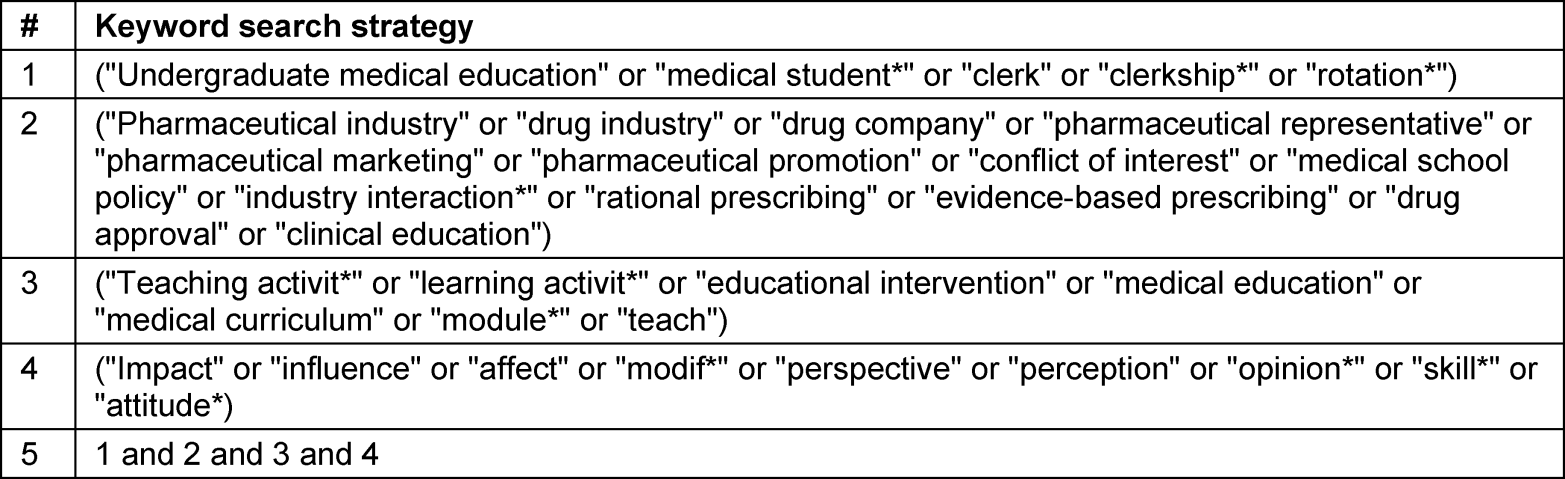
Keyword search strategy with combined search terms
