# Supplementary material for: Teaching medical students how to interact with the pharmaceutical industry: A scoping review
Source: GMS J Med Educ. 2022 Nov 15;39(5):Doc57. doi: 10.3205/zma001578 (PMC9733477; doi:10.3205/zma001578)
Supplement: Data extraction [file JME-39-57-s-001.pdf]

Attachment 1: Data extraction

Reference	Participants	Aims	Education Approach/ Intervention	Duration and frequency	Instructor's background	Kirkpatrick rating	Outcomes Assessment	Timing of post-intervention outcome measure	Main finding	Limitations
Kao et al 2011	933 (clinical) medical students from Stanford, Tufts, ECU and USC (USA) divided into graduating class (intervention) and control cohorts (penultimate year)	To assess the impact of educational interventions on medical student attitudes about pharmaceutical industry marketing	3 interventions - presentation from former pharmaceutical representative (PR), faculty debate, interactive web-based course, delivered over ~ 6 months to the intervention group, unclear if compulsory or not.	PR presentation - 1hour, faculty debate - 1hr, web-based course - completed in students' own time	PR and medical school faculty members	2a	Pre and post intervention survey regarding attitudes about pharmaceutical marketing and promotional activities	~ 1 year	More students in the inter-vention group compared to the control group held the view that pharmaceutical marketing practices exert moderate to strong in-fluence on physicians" prescribing decisions (72.2% vs 59.8%)	Incomplete baseline and follow up survey data, students not randomised into inter-vention & control groups.<br><br>The study was partly funded by a pharma-ceutical company
Sayyad et al 2017	133 2nd year (preclinical implied) medical students at BJ government medical college (India)	To evaluate the efficacy of a training program on critical appraisal of drug advertisements on the ability of students to identify breaches of guidelines and the WHO criteria for medicinal drug promotion	Students were asked to evaluate a drug promotional brochure prior to a lecture regarding critical appraisal methodology and WHO guidelines on medicinal drug promotion, followed by small group discussion. Then the same brochures were given to the students for	1hr lecture 30-minute small group discussion	Unclear	2b	Pre and post intervention survey focussed on assessing students' ability to identify violations of WHO ethical criteria for medicinal drug promotion.	Immediate and again the following year	There was a statistically significant improvement in students' ability to identify violations of guidelines on drug advertisement following the intervention	Single institution<br><br>Small sample size<br><br>No control group for comparison

			them to re-evaluate. Unclear if compulsory or not							
Wofford et al 2005	75 third year medical students (clinical) - school not mentioned, (USA).	To determine if a one-off workshop had immediate effects on the attitudes of medical students with regards to PR interactions	A mandatory discussion about the characteristics of a typical PR interaction, followed by a role play by a faculty member (MD) and a PR.	90-minute workshop.	2 faculty members (MD's) and one regional manager of pharmaceutical representatives	2a	A pre intervention survey gathered information regarding the number of previous encounters with PRs and if the student was aware about guidelines regarding such interactions. Questions regarding student attitudes towards the value of education surrounding pharmaceutical promotion, the acceptance of gifts from PRs, the level of bias in PR information and the degree of influence on prescribing patterns. The post-intervention survey included the same attitude questions as above.	Immediate	After the intervention, students were more likely to acknowledge that PR interactions influence prescribing	Single medical school affiliated with a private hospital - student opinions may differ at a medical school where there may be restrictions on the PR interactions

Wilkes et al 2001	Groups of 7 or 8 3rd year (clinical) The University of California, Los Angeles (UCLA, USA) medical students (n =144)	To assess the impact of an educational program on students' attitudes towards the accuracy and ethics of drug marketing	Pharmacist acted as a PR and delivered a presentation marketing a drug<br>- Students critiqued the presentation and had a discussion with the pharmacist who explained how their presentation reflects actual PR practice.<br>- There was then a general discussion about PR's and how to access unbiased information, compulsory.	20-minute scripted talk followed by a discussion, duration not stated	5 UCLA pharmacists, one of whom was previously a pharmaceutical representative, as well as faculty members	2a	Pre and post (after 12 weeks) intervention survey.<br>Pre intervention survey examined attitudes towards the interaction between PRs and physicians in general	12 weeks	Students' attitudes towards the ethics and accuracy of drug detailing were more uncertain following the intervention	Single institution
Shankar et al 2011	100 second year (preclinical implied) medical students at KIST medical college (Nepal)	To assess students' views on the effectiveness of a module on pharmaceutical promotion	Standardised facilitator pre-sentations, structured case scenarios, brainstorming sessions, group activities and role plays were used to explore concepts relating to the pharmaceutical industry such as marketing techniques, accepting gifts and physician-industry relationships, on-compulsory	9-hour module held over a period of ~4 months	3 MD's, one of whom is a fellow in medical education and is a pharmacologist. Occasionally students facilitated the sessions themselves, under the guidance of faculty.	1, 2a, 2b, 3	Participants gave feedback post-inter-vention regarding the effectiveness of the module itself, their level of knowledge after com-pletion of the module and their opinions regarding the importance of knowledge around this topic with re-gards to future prescribing habits	Within 2 weeks (not mentioned explicitly)	Students viewed the module to be effective in preparing them for future engagements with pharmaceutical promotion and prescribing	Only assessed students at a single institution<br>There was no pre-intervention survey available to evaluate how the intervention itself impacted students' views<br>Only 86/100 students provided the post-intervention feedback

Shankar et al 2012	100 second year (preclinical implied) medical students at KIST medical college (Nepal)	To assess the effect of an educational intervention on students' attitudes, knowledge and skills related to pharmaceutical promotion	Standardised facilitator presentations, structured case scenarios, brain-storming sessions, group activities and role plays were used to explore concepts relating to the pharmaceutical industry such as marketing techniques, accepting gifts and physician-industry relationships, non-compulsory	9-hour module held over a period of ~4 months, as above	3 MD's, one of whom is a fellow in medical education and is a pharmacologist. Occasionally students facilitated the sessions themselves, under the guidance of faculty.	2a, 2b	Retrospective pre-questionnaire and post intervention survey regarding changes in attitudes, knowledge, and skills with respect to pharmaceutical promotion, following completion of the module	Unclear, immediately after intervention implied	Students' knowledge, attitudes, and skills regarding pharmaceutical promotion 'improved' following the intervention	The retrospective pre-questionnaire administered after the intervention, as opposed to truly prior to intervention means students assessment of their attitudes etc. prior to the intervention may be inaccurate
Civaner et al 2020	2nd year (preclinical) medical students (123; intervention) and 6th year students (518; control), Bursa Uludag University (Turkey)	To assess the effectiveness of an educational program on pharmaceutical promotion strategies, such as the influence of funding on physician practice and the acceptance of gifts, with specific focus on the durability of change in students' knowledge, attitudes, and opinions	A series of lectures and small group interactive sessions which included role play and critiquing a movie about pharmaceutical promotion, non-compulsory	14 hours across 3 years	Lectures delivered by a company specialising in marketing education.<br><br>A PR participated in the role play<br><br>For other activities, instructor background unclear	2a, 2b	Pre- and post-intervention surveys were administered to assess students' opinions about physician-healthcare industry interactions. In addition, in the 4-year survey students were also asked to report the promotional methods they had been exposed to throughout their clinical years and their	Immediately after the intervention and again 4 years later	The intervention was significantly effective in the short term with measurable change in students' attitudes towards recognising the bias engendered by accepting funding from pharmaceutical companies, but these opinions did not hold 4 years later, following real-world exposure to PRs in the	Not possible to match the students' responses to the survey in their 6th year as they had forgotten the nicknames they used on the forms from 2nd year, therefore responses were compared on a whole-cohort basis (in addition to comparison to the control group_<br><br>Single institution

							opinions regarding them		clinical environment	
Corbin et al 2018	30 medical students, University of Pittsburgh, (USA). 70% 2nd year, 5%, 14% and 5% in 1st, third and fourth year respectively	To determine the impact of an educational program in increasing knowledge about pharmaceutical marketing techniques and how they impact evidence-based prescribing	A series of 6 videos, non-compulsory	2 hours	Unclear	1, 2a, 2b	Pre- and post-intervention tests were administered to assess students' knowledge and attitude on concepts surrounding pharmaceutical medicine and evidence-based prescribing. Attitudes towards the program itself were also evaluated post-intervention.	Unclear – immediately after intervention implied	There was a significant increase in students' knowledge of topics surrounding pharmaceutical medicine and evidence-based prescribing	Small sample size<br><br>Convenience sample, which may be biased towards students with greater interest in the topic of pharmaceutical industry - physician interaction
Vinson et al 1993	109 second year students, first year students (preclinical) 106 not exposed to intervention, University of Missouri-Columbia (USA)	To evaluate for changes to medical student attitudes towards the acceptance of gifts from pharmaceutical companies before and after an educational intervention	Lecture and whole-group discussion, unclear if compulsory or not	A single 50-minute lecture-discussion	'faculty'	2a	Pre (administered 6 weeks prior to lecture) and post (7 weeks after lecture) intervention survey evaluating attitudes towards acceptance of gifts from pharmaceutical companies.	6 weeks	Students were less approving of the acceptance of gifts from pharmaceutical companies following the intervention	Single institution<br>Not all students who attended the educational program responded to the surveys<br>The questionnaire results were analysed by comparing the control and intervention cohorts, as opposed to analysis of change on an individual basis<br>Students weren't randomised

										into intervention vs control<br>Not all students who participated in the lecture-discussion responded to the surveys
Markham et al 2008	243 3rd year (clinical) medical students (intervention = 120; control = 123 at Jefferson Medical College (USA))	To evaluate for changes in students' attitudes towards pharmaceutical industry and physician interactions before and after an educational program	The students were assigned articles to summarise and present to other students, followed by a discussion facilitated by a faculty member, unclear if mandatory or not	Student presentation: 20 minutes, at the beginning and end of their 6-week rotation.	Medical school faculty members	1a, 2a	Pre and post intervention survey regarding concepts surrounding physician-industry interaction, such as the acceptance of gifts, the cost of drug development, attitudes towards the amount of many pharmaceutical companies spend on marketing and other topics.	6 weeks	Students had a more cautious view towards a range of aspects of pharmaceutical industry-physician interaction, such as general attitude towards drug companies and the negative influence the acceptance of gifts can have	Single institution
Nayak et al 2011	172 2nd year medical students (preclinical implied) at Kasturba Medical College (India)	To determine the impact of teaching the critical appraisal of drug promotion according to WHO criteria	One hour lecture about WHO criteria on pharma-ceutical promotion, followed by an interactive session whereby students identified violations of the	1 hour lecture, duration of interactive session unclear	Unclear	2b	Pre-test questionnaire to identify violations of WHO criteria of a drug advertisement, post-test questionnaire with the same advertisement and questions	Immediately after intervention	There was a significant improvement in the number of students able to identify violations of guidelines and WHO criteria	Single institution<br><br>No control group<br><br>The post-test questionnaire was delivered immediately after the intervention

			criteria in different advertisements, not compulsory.							
